# Bowen’s Formula for a Dynamical Solenoid

**DOI:** 10.3390/e26110979

**Published:** 2024-11-15

**Authors:** Andrzej Biś, Wojciech Kozłowski, Agnieszka Marczuk

**Affiliations:** Faculty of Mathematics and Computer Science, University of Lodz, Banacha 22, 90-238 Łódź, Poland; wojciech.kozlowski@wmii.uni.lodz.pl (W.K.); a.namiecinska@wp.pl (A.M.)

**Keywords:** topological pressure, conformal maps, topological entropy

## Abstract

More than 50 years ago, Rufus Bowen noticed a natural relation between the ergodic theory and the dimension theory of dynamical systems. He proved a formula, known today as the Bowen’s formula, that relates the Hausdorff dimension of a conformal repeller to the zero of a pressure function defined by a single conformal map. In this paper, we extend the result of Bowen to a sequence of conformal maps. We present a dynamical solenoid, i.e., a generalized dynamical system obtained by backward compositions of a sequence of continuous surjections ${\left(f_{n}:X\to X\right)}_{n\in N}$ defined on a compact metric space (X,d). Under mild assumptions, we provide a self-contained proof that Bowen’s formula holds for dynamical conformal solenoids. As a corollary, we obtain that the Bowen’s formula holds for a conformal surjection f:X→X of a compact

## 1. Introduction and Statement of Results

In 1979, Rufus Bowen [1] noticed a natural connection between the geometric properties of a conformal repeller and the thermodynamic formalism of equation describing this repeller. Bowen proved that for a certain transformation *f* of the Riemann sphere into itself, there exists a compact *f*-invariant subset *J* called a quasi-circle. The Hausdorff dimension *t* of this quasi-circle is a unique root of the equation $P_{J}\left(-t\cdot \varphi \right)=0,$ where $P_{J}$ is the topological pressure of the map f:J→J, and $\varphi =\log(|f^{\prime }\left(z\right)|)$ is the geometric potential. In the mathematical literature, the equation $P_{J}\left(-t\cdot \varphi \right)=0$ is known as the Bowen’s formula or Bowen’s equation. In 1982, Ruelle [2] showed that the Hausdorff dimension of the Julia set of a uniformly hyperbolic rational map depends real-analytically on parameters. The Bowen’s equation has various generalizations and applications in both real and complex dynamics. For example, the result of Barański [3] for some hyperbolic meromorphic maps was generalized by Kotus and Urbański [4] to the case of so-called regular Walters expanding conformal maps. The thermodynamical formalism theory for hyperbolic maps in the exponential family was developed by Urbański and Zdunik [5,6]. Also there is a recent paper by Barański, Karpińska, and Zdunik [7] on Bowen’s formula for meromorphic functions. There are also known generalizations of Bowen’s formula to the case of semigroup; Jaerisch and Sumi [8] obtained Bowen’s formula for a pre-Julia set of a nicely expanding rational semigroups. In [9,10], one can find applications of Bowen’s equation in examples for expanding Markov maps. In 2008, Rugh [11] generalized Bowen’s result and proved the following result.

**Theorem** **1** (Rugh [11]). *Let M be a Riemannian manifold, V⊂M be an open set, f:V→M be a conformal map of class $C^{1+\epsilon },$ and J⊂V be its repeller. Then, the equation*

$$P_{J}\left(-t\cdot \varphi \right)=0,$$

where φ(x)=log(||Df(x)||), has a unique root $t^{*}$ equal to the Hausdorff dimension of the set J.

In 2011, Climenhaga [12] presented a generalization of the Bowen’s formula to the case of a continuous map f:X→X defined on a compact metric space (X,d) with a nonzero dilatation, denoted here by $a_{w}\left(x\right).$ In Climenhaga’s work, we encounter the following definition of conformality. A map f:X→X is *conformal with dilatation* $a_{w}\left(x\right)$ if for any x∈X there exists a limit

$$a_{w}\left(x\right):=\lim_{y\to x}\frac{d(f(x),f(y))}{d(x,y)}$$

and the map $a_{w}:X\to \left[0,\infty \right)$ is continuous. However, this notion is not suitable for our approach. Therefore, in Section 2, we introduce and apply a notion of strong dilatation and strong conformality.

For a conformal map (respectively, for strong conformal map), we define the Birkhoff sum, lower and upper Lyapunov exponents as follows.

$$\begin{array}{cc} S_{n}\left(\log a_{w}\right)\left(x\right) & =\sum_{k=0}^{n-1}\log a_{w}\left(f^{k}\left(x\right)\right),\\ \lambda_{n}\left(x\right) & =\frac{1}{n}\cdot S_{n}\left(\log a_{w}\right)\left(x\right). \end{array}$$

The limits

$$\underline{\lambda }\left(x\right)=\underset{n\to \infty }{lim inf}\lambda_{n}\left(x\right)$$

are called the lower Lyapunov exponents, and

$$\bar{\lambda }\left(x\right)=\underset{n\to \infty }{lim sup}\lambda_{n}\left(x\right)$$

are the upper Lyapunov exponents. For a subset E⊂R, we denote by

$$A\left(E\right)=\{x\in X:\left[\underline{\lambda }\left(x\right),\bar{\lambda }\left(x\right)\right]\subset E\}.$$

Climenhaga introduced the set B as the set of points x∈X for which the condition

$$\inf\{S_{n-k}\left(\log a_{w}\right)\left(f^{k}\left(x\right)\right)+n\cdot \varepsilon :0\leq k\leq n,n\in N\}>-\infty$$

holds, and he proved the following theorem.

**Theorem** **2** (Climenhaga [12]). Let X be a compact metric space, and f:X→X be a continuous conformal map with dilatation $a_{w}\left(x\right).$ Assume that f has no points of zero or infinite dilatation, meaning that for any x∈X, the inequalities $0<a_{w}\left(x\right)<\infty$ hold. Then, for any subset Z⊂A(0,∞)∩B, the unique root $t^{*}$ of the pressure function $t\mapsto P_{Z}\left(-t\cdot \log a_{w}\right)$ is equal to the Hausdorff dimension $dim_{H}\left(Z\right)$ of Z.

The rest of this section is devoted to the Bowen’s formula for a dynamical solenoid, i.e., the sequence $f_{\infty }={\left(f_{n}:X\to X\right)}_{n\in N}$ of continuous surjections defined on a compact metric space (X,d). In general, a sequence $f_{\infty }={\left(f_{n}:X\to X\right)}_{n\in N}$ of continuous surjections determines two distinct dynamical systems: nonautonomous dynamical system (when we consider forward compositions of $f_{n}:X\to X$) and dynamical solenoid (when we consider dynamics determined by inverse limit of the sequence). There are many papers on the dynamics of nonautonomous dynamical systems. The dynamics of dynamical solenoids, which are equally interesting, were studied less intensively (see, for example, [13,14]).

Additionally, we assume that $f_{\infty }$ is expanding and strongly conformal; it means that each $f_{n}:X\to X$ is a strongly conformal and expanding map (for precise definitions, see Section 2).

We also refer the reader to Section 3 where they will find detailed definitions of the sequence $a_{\infty }$, Lyapunov exponents $\underline{\lambda^{f_{\infty }}},\bar{\lambda^{f_{\infty }}}$ and A(E) in our case.

Our proof of the Bowen’s formula for a dynamical solenoid is inspired by Climenhaga’s paper, where he considered a continuous conformal map on a compact metric space. The main results of the paper are the following two theorems. In Theorem 3, we consider a topological entropy $h_{top}\left(f_{\infty },Z\right)<\infty$ of a dynamical solenoid $f_{\infty },$ restricted to a subset Z⊂X, and provide estimation of a unique root of its pressure function $t\to P_{Z}\left(-t\log a_{\infty }\right)$.

**Theorem** **3.** *Assume that $h_{top}\left(f_{\infty },Z\right)<\infty$. Let c,C∈R with c≤C, be such that for any x∈Z,*

$$c\leq \underline{\lambda^{f_{\infty }}}\left(x\right)\leq \bar{\lambda^{f_{\infty }}}\left(x\right)\leq C.$$

*If c>0 or C<0, then there exists a unique $t^{\prime }\in \left[\frac{1}{C}h_{top}\left(f_{\infty },Z\right),\frac{1}{c}h_{top}\left(f_{\infty },Z\right)\right]$, such that $P(-t^{\prime }\log a_{\infty })=0$. In particular, when c=C≠0, i.e., the Lyapunov exponent $\lambda^{f_{\infty }}\neq 0$ is constant on Z, then*

$$t^{\prime }=\frac{h_{top}\left(f_{\infty },Z\right)}{\lambda^{f_{\infty }}}.$$

*In this case,*

$$P_{Z}\left(-\frac{h_{top}\left(f_{\infty },Z\right)}{\lambda^{f_{\infty }}}\log a_{\infty }\right)=0.$$

In case of a dynamical conformal simple and expanding solenoid, called for simplicity a DCSE-solenoid (see Section 5), we show that the Bowen’s formula holds, i.e., we prove the following result.

**Theorem** **4.** Let $\left(X_{\infty },f_{\infty },a_{\infty }\right)$ be a DCSE-solenoid defined on a compact metric space (X,d), and let Z⊂X. Assume that $h_{top}\left(f_{\infty },Z\right)<\infty$ and Z⊂A((α,∞)) for some α>0. Then, there exists a unique root $t^{*}$ of the pressure function $t\mapsto P_{Z}\left(-t\cdot \log a_{\infty }\right))$ with $\dim_{H}\left(Z\right)=t^{*}$.

**Corollary** **1.** Let X be a compact metric space and f:X→X be a strong conformal surjection with strong dilatation a. If f is regular and locally expanding, i.e., 1<a(x)<∞ for any x∈X, then, for any subset Z⊂A(α,∞), where 0<α<∞, the unique root $t^{*}$ of the pressure function $t\mapsto P_{Z}\left(-t\cdot \log\left(a\right)\right)$ is equal to the Hausdorff dimension $dim_{H}\left(Z\right)$ of Z.

The paper is organized as follows. In the Introduction, we provide the motivation of our research, the history of Bowen’s formula, and present main results of the paper. Section 2 is devoted to strong conformal maps and strong dilatation and basic properties of these notions. In Section 3, we introduce a notion of Lyapunov exponent, topological entropy, and topological pressure (in the spirit of Carathéodory structures elaborated by Pesin [15]) of a conformal dynamical solenoid. We prove basic properties of a pressure function of a strongly conformal dynamical solenoid. We also present a proof of Theorem 3. In Section 4, we describe the equivalence of two different approaches to the Hausdorff dimension. Section 5 is devoted to the proof of Lemma 11, the essential lemma used in the proof of Theorem 4. In Section 6, we prove Theorem 4. Finally, in the Section 7, we present an example showing that the notions of conformal map and of strongly conformal map are not equivalent.

## 2. Conformal Mappings

Let (X,d) be a metric space. For any x∈X and δ>0, let B(x,δ) denote the open ball centered at point *x* with radius δ. We will say that the mapping f:X→X is *strong conformal* if there exists a continuous function a:X→[0,∞], called a *strong dilatation*, such that for any $x_{0}\in X$,

(1) $$\lim_{\begin{array}{c} x\to x_{0}\\ y\to x_{0}\\ x\neq y \end{array}}\frac{d(f(y),f(x))}{d(y,x)}=a\left(x_{0}\right).$$

If, for any x∈X, 0<a(x)<∞, we call *f* a strong conformal regular mapping.

**Lemma** **1.** *If f:X→X is a strong conformal mapping with strong dilatation a<∞, then f is locally Lipschitz, i.e., for any $x_{0}\in X$, there exists δ>0 and a constant L such that*

$$d\left(f\left(x\right),f\left(y\right)\right)\leq Ld\left(x,y\right),\;x,y\in B\left(x_{0},\delta \right).$$

*In particular, f is continuous.*

**Proof.**  Let $x_{0}\in X$. Since $a(x_{0})<\infty$ and (1), there exists δ>0 such that for $x,y\in B(x_{0},\delta )$, the inequality d(f(y),f(x))<L·d(y,x) is satisfied with $L=a(x_{0})+1$. This completes the proof. □

**Corollary** **2.** *If f:X→X is a strong conformal mapping with strong dilatation 0<a<∞ and X is a compact metric space, then f is Lipschitz on X. In particular, f is uniformly continuous.*

**Proof.**  This follows directly by Lemma 1 and the fact that any locally Lipschitz function is Lipschitz on any compact set. □

**Lemma** **2.** *Assume that f:X→X is a strong conformal mapping with strong dilatation a. Let $x_{0}\in X$. If $a(x_{0})>0$, then there exists ε>0 such that f(y)≠f(x) whenever $x,y\in B(x_{0},\varepsilon )$ and y≠x. In other words, f is a one-to-one mapping in some neighborhood of the point $x_{0}$.*

**Proof.**  Let $A\in (0,a\left(x_{0}\right))$. Such *A* exists due to a(x)>0. From the condition (1), it follows that there exists ε>0 such that

d(f(y),f(x))>A·d(y,x)>0

for any $x,y\in B(x_{0},\varepsilon )$ and y≠x. □

**Corollary** **3.** *Every strong regular conformal mapping is continuous and locally one-to-one.*

**Proposition** **1.** *Let f:X→X be a strong regular conformal mapping defined on a compact metric space (X,d), and let a denote the strong dilatation of f. Then f is uniformly locally one-to-one, i.e., there exists $\hat{\delta }>0$ such that for any U⊂X with diameter $<\hat{\delta }$, f|U is one-to-one.*

**Proof.**  By Corollary 3 there exists a finite cover $U=\{U_{1},\ldots ,U_{n}\}$ of *X*, such that for any i=1,…,n, $f|U_{i}$ is one-to-one. Let $\hat{\delta }$ be the Lebesgue number of the cover U. Then, for any set *U* with diameter $<\hat{\delta }$, $U\subset U_{i}$ for some *i*. Consequently, f|U is one-to-one. □

**Lemma** **3.** *Assume that $f_{i}:X\to X$, i=1,2, are strong regular conformal mappings with strong dilatations $a_{i}$. Then, the composition $f_{1}\circ f_{2}:X\to X$ is a continuous strong regular conformal mapping with strong dilalation $a=\left(a_{1}\circ f_{2}\right)\cdot a_{2}$, i.e., $a\left(x_{0}\right)=a_{1}\left(f_{2}\left(x_{0}\right)\right)\cdot a_{2}\left(x_{0}\right).$*

**Proof.**  Notice that $f_{1}\circ f_{2}:X\to X$ is continuous as the composition of continuous mappings. Let $x_{0}\in X$ be fixed. By Lemma 2, $f_{2}$ is one-to-one in some neighborhood of $x_{0}$. Thus, we obtain

$$\begin{array}{ccc} & & \lim_{\begin{array}{c} x\to x_{0}\\ y\to x_{0}\\ x\neq y \end{array}}\frac{d(f_{1}\left(f_{2}\left(y\right)\right),f_{1}\left(f_{2}\left(x\right)\right))}{d(y,x)}\\ & = & \lim_{\begin{array}{c} x\to x_{0}\\ y\to x_{0}\\ x\neq y \end{array}}\frac{d(f_{1}\left(f_{2}\left(y\right)\right),f_{1}\left(f_{2}\left(x\right)\right))}{d(f_{2}\left(y\right),f_{2}\left(x\right))}\cdot \frac{d(f_{2}\left(y\right),f_{2}\left(x\right))}{d(y,x)}\\ & = & \lim_{\begin{array}{c} x\to x_{0}\\ y\to x_{0}\\ x\neq y \end{array}}\frac{d(f_{1}\left(f_{2}\left(y\right)\right),f_{1}\left(f_{2}\left(x\right)\right))}{d(f_{2}\left(y\right),f_{2}\left(x\right))}\cdot \lim_{\begin{array}{c} x\to x_{0}\\ y\to x_{0}\\ x\neq y \end{array}}\frac{d(f_{2}\left(y\right),f_{2}\left(x\right))}{d(y,x)}\\ & = & a_{1}\left(f_{2}\left(x_{0}\right)\right)\cdot a_{2}\left(x_{0}\right). \end{array}$$

□

**Corollary** **4.** *If $f_{i}$, i=1,…,N, are continuous strong conformal mappings with strong dilatations $a_{i}$, then the composition $f_{1}\circ f_{2}\circ \cdots \circ f_{N}$ is strong conformal with strong dilatation*

$$a=\left(a_{1}\circ f_{2}\circ \cdots \circ f_{N}\right)\cdot \left(a_{2}\circ f_{3}\circ \cdots \circ f_{N}\right)\cdots \left(a_{N-1}\circ f_{N}\right)\cdot a_{N}.$$

Equip the Cartesian product X×X with the product metric $\tilde{d}$ defined by

$$\tilde{d}\left(\left(x_{1},y_{1}\right),\left(x_{2},y_{2}\right)\right)=d\left(x_{1},x_{2}\right)+d\left(y_{1},y_{2}\right).$$

The diagonal is defined as Δ={(x,x):x∈X}⊂X×X. The diagonal is a closed subset of X×X, and, in particular, Δ is compact if *X* is compact. By a neighborhood of the diagonal Δ, we mean any open subset U⊃Δ in X×X.

**Lemma** **4.** *Assume that (X,d) is a compact metric space. Let U be a neighborhood of the diagonal Δ⊂X×X. There exists $\delta =\delta_{U}>0$ such that for any x,y∈X,*

(x,y)∈Uwheneverd(x,y)<δ.

**Proof.**  Due to the compactness of the diagonal Δ, there exists δ>0 such that (x,y)∈U whenever $\tilde{d}\left(\left(x,y\right),\left(x,x\right)\right)<\delta$. From the definition of $\tilde{d}$, we have

$$\tilde{d}\left(\left(x,y\right),\left(x,x\right)\right)=d\left(x,x\right)+d\left(x,y\right)=0+d\left(x,y\right)=d\left(x,y\right).$$

This completes the proof. □

**Proposition** **2.** *Assume that f:X→X is a strong regular conformal mapping with a strong dilatation a. The function ${\tilde{a}}_{f}:X\times X\to R$,*

$${\tilde{a}}_{f}\left(x,y\right)=\left\{\begin{array}{cc} \frac{d(f(x),f(y)}{d(x,y)}, & \mathrm{when}\;x\neq y\\ a(x), & \mathrm{when}\;x=y, \end{array}\right.$$

*is continuous. In particular, ${\tilde{a}}_{f}$ is uniformly continuous when X is compact. Moreover, if X is compact, ${\tilde{a}}_{f}$ is positive in some neighborhood of the diagonal Δ⊂X×X.*

**Proof.**  The continuity of *f* implies the continuity of the function ${\tilde{a}}_{f}$ at every point (x,y)∈X×X where x≠y. Let $x_{0}\in X$ and ε>0. From the conformality condition, there exists $\delta_{1}>0$ such that $|{\tilde{a}}_{f}\left(x,y\right)-a\left(x_{0}\right)|<\varepsilon$, when x≠y, $d\left(x,x_{0}\right)<\delta_{1}$, and $d\left(y,x_{0}\right)<\delta_{1}$. Since *a* is continuous, there exists $\delta_{2}>0$ such that $|{\tilde{a}}_{f}\left(x,x\right)-a\left(x_{0}\right)|=|a\left(x\right)-a\left(x_{0}\right)|<\varepsilon$, when $d\left(x,x_{0}\right)<\delta_{2}$. Consequently, for $\delta =\min\{\delta_{1},\delta_{2}\}$,

$$|{\tilde{a}}_{f}\left(x,y\right)-a\left(x_{0}\right)|<\varepsilon ,\;\mathrm{when}\;d\left(x,x_{0}\right)<\delta ,\;\;d\left(y,x_{0}\right)<\delta .$$

The definition of the metric $\tilde{d}$ yields

$$|{\tilde{a}}_{f}\left(x,y\right)-a\left(x_{0}\right)|<\varepsilon ,\;\mathrm{when}\;\tilde{d}\left(\left(x,y\right),\left(x_{0},y_{0}\right)\right)<\delta .$$

This proves the continuity of the function ${\tilde{a}}_{f}$. Since a>0, ${\tilde{a}}_{f}$ is positive in some neighborhood of Δ. □

## 3. Dynamical Solenoids

Let (X,d) be a (compact) metric space. For any natural number n≥0, let $X_{n}=X$. Consider $X_{\infty }={\left(X_{n}\right)}_{n\geq 0}$, and let $f_{\infty }$ denote the sequence of continuous surjections $f_{n}:X_{n}\to X_{n-1}$ for n≥1. The system $\left(X_{\infty },f_{\infty }\right)$ is called a *dynamical solenoid*. If all elements of the sequence $f_{\infty }$ are strong conformal mappings, we place $a_{\infty }={\left(a_{n}\right)}_{n\geq 1},$ where $a_{n}$ denotes the strong dilatation of $f_{n}$. If the sequence $f_{\infty }$ consists of strong regular conformal mappings, then $\left(X_{\infty },f_{\infty },a_{\infty }\right)$ is called a *regular dynamical conformal solenoid*.

For any integers 0≤i<j, define

$$f_{i,j}=f_{i+1}\circ \cdots \circ f_{j}:X_{j}⟶X_{i}.$$

Clearly, $f_{j-1,j}=f_{j}:X_{j}\to X_{j-1}$. Also, for all integers j≥0, let $f_{j,j}={\mathrm{id}}_{X_{j}}$. It follows from the definitions that for any integers 0≤i≤k≤j

$$f_{i,k}\circ f_{k,j}=f_{i,j}.$$

In the case when $\left(X_{\infty },f_{\infty },a_{\infty }\right)$ is a strong regular dynamical conformal solenoid, by Corollary 4, for 0≤i<j, each $f_{i,j}$ is a strong regular conformal mapping with strong dilatation

$$\begin{array}{ccc} a_{i,j} & = & a_{i+1}\circ f_{i+1,j}\cdot a_{i+2}\circ f_{i+2,j}\cdots a_{j-1}\circ f_{j-1,j}\cdot a_{j}\circ f_{j,j}\\ & = & \prod_{k=1}^{j-i}a_{i+k}\circ f_{i+k,j}. \end{array}$$

Moreover, we can set $a_{i,i}=1$, which is consistent with the fact that $f_{i,i}$ is the identity mapping on $X_{i}$, and, thus, conformal with unit dilatation.

For x∈X, n≥0, and δ>0, the *n*-th dynamical ball $B\left(x,n,\delta \right)\subset X_{n}=X$ is defined as follows

$$\begin{array}{ccc} B(x,n,\delta ) & = & \left\{y\in X_{n}:d\left(f_{i,n}\left(x\right),f_{i,n}\left(y\right)\right)<\delta ,\;\;\forall_{1\leq i\leq n}\right\}\\ & = & \overset{n}{\underset{i=0}{⋂}}{f_{i,n}}^{-1}\left(B(f_{i,n}\left(x\right),\delta )\right). \end{array}$$

Since,

$${f_{n,n}}^{-1}\left(B(f_{n,n}\left(x\right),\delta )\right)=B\left(x,\delta \right),$$

for any n≥0, we have

(2) B(x,n,δ)⊂B(x,δ)andB(x,0,δ)=B(x,δ).

Let us assume that a sequence of continuous functions $\varphi_{\infty }={\left(\varphi_{n}:X_{n}\to R\right)}_{n\geq 1}$ is given, which we will call a *multipotential*. For n≥1, we define

$$\begin{array}{ccc} S_{n}^{f_{\infty }}\varphi_{\infty } & = & \sum_{i=1}^{n}\varphi_{i}\circ f_{i,n}:X_{n}\to R. \end{array}$$

### 3.1. Lyapunov Exponents for Dynamical Solenoids

In the case when $\left(X_{\infty },f_{\infty },a_{\infty }\right)$ is a regular dynamical conformal solenoid, for n≥1, we set

$$\begin{array}{ccc} \lambda_{n}^{f_{\infty }} & = & \log\left(\sqrt[{n}]{a_{0,n}}\right)=\frac{1}{n}\log\left(\prod_{k=1}^{n}a_{k}\circ f_{k,n}\right)\\ & = & \frac{1}{n}\sum_{k=1}^{n}\left(\log a_{k}\right)\circ f_{k,n}=\frac{1}{n}S_{n}^{f_{\infty }}\left(\log a_{\infty }\right). \end{array}$$

More precisely, for $x\in X_{n}$, the function $\lambda_{n}^{f_{\infty }}:X_{n}\to R$, is defined by

$$\lambda_{n}^{f_{\infty }}\left(x\right)=\frac{1}{n}S_{n}^{f_{\infty }}\left(\log a_{\infty }\right)\left(x\right)=\frac{1}{n}\sum_{k=1}^{n}\log a_{k}\left(f_{k,n}\left(x\right)\right).$$

For 1≤i≤j, we also define

$$\begin{array}{ccc} S_{i,j}^{f_{\infty }}\left(\log a_{\infty }\right)\left(x\right) & = & \sum_{k=i}^{j}\log a_{k}\left(f_{k,j}\left(x\right)\right)\\ \lambda_{i,j}^{f_{\infty }}\left(x\right) & = & \frac{1}{j-i+1}S_{i,j}^{f_{\infty }}\left(\log a_{\infty }\right)\left(x\right). \end{array}$$

Note that

$$\begin{array}{ccc} S_{j,j}^{f_{\infty }}\left(\log a_{\infty }\right) & = & \log a_{j},\;S_{1,n}^{f_{\infty }}=S_{n}^{f_{\infty }},\\ \lambda_{j,j}^{f_{\infty }} & = & \log a_{j},\;\lambda_{1,n}^{f_{\infty }}=\lambda_{n}^{f_{\infty }}. \end{array}$$

Let x∈X. Without any additional assumptions, there is no reason for the sequence $\left(\lambda_{n}^{f_{\infty }}\left(x\right)\right)$ to converge. However, there exist the upper limit $\bar{\lambda^{f_{\infty }}}\left(x\right)$ and the lower limit $\underline{\lambda^{f_{\infty }}}\left(x\right)$, called, respectively, the *upper* and *lower Lyapunov exponents*. More precisely,

$$\bar{\lambda^{f_{\infty }}}\left(x\right)=\underset{n\to \infty }{lim sup}\lambda_{n}^{f_{\infty }}\left(x\right),\;\underline{\lambda^{f_{\infty }}}\left(x\right)=\underset{n\to \infty }{lim inf}\lambda_{n}^{f_{\infty }}\left(x\right).$$

In the case where the sequence $\left(\lambda_{n}^{f_{\infty }}\left(x\right)\right)$ converges, its limit is called the *Lyapunov exponent* and is denoted by $\lambda^{f_{\infty }}\left(x\right)$, i.e.,

$$\lambda^{f_{\infty }}\left(x\right)=\lim_{n\to \infty }\lambda_{n}^{f_{\infty }}\left(x\right).$$

For any E⊂R, let

$$A\left(E\right)=\{x\in X:\left[\underline{\lambda^{f_{\infty }}}\left(x\right),\bar{\lambda^{f_{\infty }}}\left(x\right)\right]\subset E\}$$

In particular, when E={α}, we write A(α). In this case,

$$A\left(\alpha \right)=\{x\in X:\lambda^{f_{\infty }}\left(x\right)=\alpha \}.$$

### 3.2. Topological Pressure for Dynamical Solenoids

Let Z⊂X. For each natural number N≥1 and each real number δ>0, consider the family C(Z,N,δ) of all dynamical balls B(x,n,δ) such that x∈Z and n≥N. Since *X* is compact, there is a finite or countable set of balls $\left\{B\left(x_{i},n_{i},\delta \right)\right\}\subset C\left(Z,N,\delta \right)$ indexed by pairs $\left(x_{i},n_{i}\right)$, which forms an open cover of the set *Z*. We denote the family of all such countable covers by P(Z,N,δ). We denote the family of all sets $\left\{\left(x_{i},n_{i}\right)\right\}$ indexing these covers by I(Z,N,δ), i.e.,

$$\left\{\left(x_{i},n_{i}\right)\right\}\in I\left(Z,N,\delta \right)\;\;\Leftrightarrow \;\;\left\{B\left(x_{i},n_{i},\delta \right)\right\}\in P\left(Z,N,\delta \right).$$

Let s∈R. For $I=\left\{\left(x_{i},n_{i}\right)\right\}\in I\left(Z,N,\delta \right)$ and t∈R, we define

$$\begin{array}{cc} \; & \Phi \left(s,I\right)=\Phi \left(s,I,\varphi_{\infty }\right)=\sum_{(x_{i},n_{i})\in I}\exp\left(-n_{i}s+\left(S_{n_{i}}^{f_{\infty }}\varphi_{\infty }\right)\left(x_{i}\right)\right),\\ \; & \Phi \left(s,I,t\right)=\Phi \left(s,I,t,\varphi_{\infty }\right)=\sum_{(x_{i},n_{i})\in I}\exp\left(-n_{i}s+t\left(S_{n_{i}}^{f_{\infty }}\varphi_{\infty }\right)\left(x_{i}\right)\right). \end{array}$$

Note that Φ(s,I),Φ(s,I,t)∈(0,∞] and Φ(s,I)=Φ(s,I,1). Directly from the definition of the operator $S_{n}^{f_{\infty }}$, it follows that

$$\Phi \left(s,I,t\varphi_{\infty }\right)=\Phi \left(s,I,t,\varphi_{\infty }\right).$$

Note that every cover belonging to the family P(Z,N+1,δ), belongs to P(Z,N,δ), i.e.,

(3) P(Z,N+1,δ)⊂P(Z,N,δ).

As a consequence, we obtain the inclusion

(4) I(Z,N+1,δ)⊂I(Z,N,δ).

We define

$$\begin{array}{ccc} m_{P}\left(Z,s,\varphi_{\infty },N,\delta \right) & = & \inf\left\{\Phi \left(s,I,\varphi_{\infty }\right):I\in I\left(Z,N,\delta \right)\right\}, \end{array}$$

which has values in [0,∞].

**Lemma** **5.** *The function*

$$N\mapsto m_{P}\left(Z,s,\varphi_{\infty },N,\delta \right),\;N\in \left\{1,2,3,\ldots \right\}$$

*is nondecreasing.*

**Proof.**  A direct consequence of the inclusion (4) and the properties of the lower bound. □

By Lemma 5, it follows that there exists a limit

$$m_{P}\left(Z,s,\varphi_{\infty },\delta \right)=\lim_{N\to \infty }m_{P}\left(Z,s,\varphi_{\infty },N,\delta \right)\in \left[0,\infty \right].$$

This limit is equal to

$$\sup\left\{m_{P}\left(Z,s,\varphi_{\infty },N,\delta \right):N\geq 1\right\}\in \left[0,\infty \right].$$

**Lemma** **6.** *The functions*

$$\begin{array}{ccc} s & \mapsto & m_{P}\left(Z,s,\varphi_{\infty },N,\delta \right),\\ s & \mapsto & m_{P}\left(Z,s,\varphi_{\infty },\delta \right),\;\;where\;s\in R, \end{array}$$

*are nonincreasing.*

**Proof.**  Let t≥s. Then, for any integer n≥1, $e^{-ns}\geq e^{-nt}$. Therefore, using the definition of Φ(s,I), we have

$$\begin{array}{cc} \Phi (s,I) & =\sum_{(x_{i},n_{i})\in I}\exp\left(-n_{i}s+\left(S_{n_{i}}^{f_{\infty }}\varphi_{\infty }\right)\left(x_{i}\right)\right)\\ \; & =\sum_{(x_{i},n_{i})\in I}e^{-n_{i}s}\exp\left(\left(S_{n_{i}}^{f_{\infty }}\varphi_{\infty }\right)\left(x_{i}\right)\right)\\ \; & \geq \sum_{(x_{i},n_{i})\in I}e^{-n_{i}t}\exp\left(\left(S_{n_{i}}^{f_{\infty }}\varphi_{\infty }\right)\left(x_{i}\right)\right)\\ \; & =\sum_{(x_{i},n_{i})\in I}\exp\left(-n_{i}t+\left(S_{n_{i}}^{f_{\infty }}\varphi_{\infty }\right)\left(x_{i}\right)\right)=\Phi \left(t,I\right). \end{array}$$

Therefore, we have

$$\begin{array}{ccc} \Phi (s,I) & \geq & \Phi \left(t,I\right)\geq \inf\left\{\Phi (t,I):I\in I(Z,N,\delta )\right\}\\ & = & m_{P}\left(Z,t,\varphi_{\infty },N,\delta \right). \end{array}$$

It follows that

$$m_{P}\left(Z,s,\varphi_{\infty },N,\delta \right)\geq m_{P}\left(Z,t,\varphi_{\infty },N,\delta \right),\;\mathrm{for}\;t\geq s,$$

which proves that the first of the functions in the formulation of the lemma is nonincreasing. Next, by passing to the limit in the above inequality with N→∞, we obtain

$$m_{P}\left(Z,s,\varphi_{\infty },\delta \right)\geq m_{P}\left(Z,t,\varphi_{\infty },\delta \right),\;\mathrm{for}\;t\geq s,$$

which proves that the second of the functions in the formulation of the lemma is nonincreasing.

□

**Lemma** **7.** *The functions*

$$\begin{array}{ccc} \delta & \mapsto & m_{P}\left(Z,s,\varphi_{\infty },N,\delta \right),\\ \delta & \mapsto & m_{P}\left(Z,s,\varphi_{\infty },\delta \right),\;where\;\delta >0, \end{array}$$

*are nonincreasing.*

**Proof.**  Let η≥δ>0. Then, B(x,n,δ)⊂B(x,n,η). Hence, I(Z,N,δ)⊂I(Z,N,η) and, therefore,

$$\begin{array}{ccc} m_{P}\left(Z,s,\varphi_{\infty },N,\delta \right) & = & \inf\{\Phi (s,I):I\in I(Z,N,\delta )\}\\ & \geq & \inf\{\Phi (s,I):I\in I(Z,N,\eta )\}\\ & = & m_{P}\left(Z,s,\varphi_{\infty },N,\eta \right), \end{array}$$

which proves the monotonicity of the first function in the lemma. Next, by passing to the limit in the above inequality with N→∞, we obtain the monotonicity of the second function. □

**Corollary** **5.**

(a) *The function*
$$\left(s,N,\delta \right)\mapsto m_{P}\left(Z,s,\varphi_{\infty },N,\delta \right),$$
  *where (s,N,δ)∈R×{1,2,…}×(0,∞), is nonincreasing with respect to s and δ, and nondecreasing with respect to N.*
(b) *The function*
$$\left(s,\delta \right)\mapsto m_{P}\left(Z,s,\varphi_{\infty },\delta \right),$$
  *where (s,δ)∈R×(0,∞), is nonincreasing with respect to s and δ.*

**Lemma** **8.** *Let t>s. Then,*

(a) *If $m_{P}\left(Z,s,\varphi_{\infty },\delta \right)<\infty$, then $m_{P}\left(Z,t,\varphi_{\infty },\delta \right)=0$.*
(b) *If $m_{P}\left(Z,t,\varphi_{\infty },\delta \right)>0$, then $m_{P}\left(Z,s,\varphi_{\infty },\delta \right)=\infty$.*

**Proof.**  Notice that for I∈I(Z,N,δ),

(5) $$\Phi \left(t,I\right)\leq e^{-N(t-s)}\cdot \Phi \left(s,I\right),$$

or, equivalently,

(6) $$\Phi \left(s,I\right)\geq e^{N(t-s)}\cdot \Phi \left(t,I\right).$$

(a) Using (5), we obtain the following:

$$\begin{array}{cc} m_{P}\left(Z,t,\varphi_{\infty },N,\delta \right) & =\inf\left\{\Phi (t,I):I\in I(Z,N,\delta )\right\}\\ \; & \leq \Phi \left(t,I\right)\leq e^{-N(t-s)}\cdot \Phi \left(s,I\right),\\ m_{P}\left(Z,t,\varphi_{\infty },N,\delta \right) & \leq e^{-N(t-s)}\cdot \inf\left\{\Phi (s,I):I\in I(Z,N,\delta )\right\}\\ \; & =e^{-N(t-s)}\cdot m_{P}\left(Z,s,\varphi_{\infty },N,\delta \right)\\ \; & \leq e^{-N(t-s)}\cdot \sup\left\{m_{P}\left(Z,s,\varphi_{\infty },N,\delta \right):N\geq 1\right\}\\ \; & =e^{-N(t-s)}\cdot m_{P}\left(Z,s,\varphi_{\infty },\delta \right) \end{array}$$

Since t−s>0, $\lim_{N\to \infty }e^{-N(t-s)}=0$. Consequently, assuming that $m_{P}\left(Z,s,\varphi_{\infty },\delta \right)<\infty$, we obtain

$$\begin{array}{cc} m_{P}\left(Z,t,\varphi_{\infty },\delta \right) & =\lim_{N\to \infty }m_{P}\left(Z,t,\varphi_{\infty },N,\delta \right)\\ \; & \leq m_{P}\left(Z,s,\varphi_{\infty },\delta \right)\cdot \lim_{N\to \infty }e^{-N(t-s)}=0, \end{array}$$

which proves (a).

The proof of (b) is analogous to the proof of item (a). □

The function $s\mapsto m_{P}\left(Z,s,\varphi_{\infty },\delta \right)$ has a unique critical point which is denoted by $P_{Z}\left(\varphi_{\infty },\delta \right)$ (by Lemma 8).

More precisely,

(7) $$\begin{array}{ccc} P_{Z}\left(\varphi_{\infty },\delta \right) & = & \sup\{s\in R:m_{P}\left(Z,s,\varphi_{\infty },\delta \right)=\infty \}\\ & = & \inf\{s\in R:m_{P}\left(Z,s,\varphi_{\infty },\delta \right)=0\}. \end{array}$$

Thus, by Lemma 7, it follows that there exists a limit:

(8) $$P_{Z}\left(\varphi_{\infty }\right)=\lim_{\delta \to 0^{+}}P_{Z}\left(\varphi_{\infty },\delta \right)=\sup\left\{P_{Z}\left(\varphi_{\infty },\delta \right):\delta >0\right\}.$$

The limit $P_{Z}\left(\varphi_{\infty }\right)$ is called the *topological pressure* of the dynamical solenoid with respect to the subset Z⊂X and the multipotential $\varphi_{\infty }$.

**Lemma** **9.** *If $Z_{1}\subset Z_{2}\subset X$, then $P_{Z_{1}}\left(\varphi_{\infty }\right)\leq P_{Z_{2}}\left(\varphi_{\infty }\right).$*

**Proof.**  It easily follows from the definition and the properties stated above. □

As a corollary, we obtain the following lemma.

**Lemma** **10.** *If ${\left(Z_{m}\right)}_{m\in N}$ is a sequence of subsets of the space X such that ${⋃}_{m\in N}Z_{m}=Z$ and $Z_{m}\subset Z_{m+1}$, then*

$$P_{Z}\left(\varphi_{\infty }\right)=\underset{m\in N}{\sup}P_{Z_{m}}\left(\varphi_{\infty }\right)=\lim_{m\to \infty }P_{Z_{m}}\left(\varphi_{\infty }\right).$$

### 3.3. Topological Entropy and Pressure for Dynamical Solenoids

Recall the definition of topological entropy $h_{top}\left(f_{\infty },Z\right)$ restricted to a set Z⊂X. Define

$$m_{h}^{f_{\infty }}\left(Z,t,N,\delta \right)=\inf\left\{\sum_{\begin{array}{c} (x_{i},n_{i})\in I \end{array}}\exp\left(-n_{i}\cdot t\right):\right.:\left.Z\subset \underset{\begin{array}{c} (x_{i},n_{i})\in I \end{array}}{⋃}B\left(x_{i},n_{i},\delta \right),\;n_{i}\geq N,\;x_{i}\in Z\right\}.$$

Then, we have the following relations:

$$\begin{array}{ccc} m_{h}^{f_{\infty }}\left(Z,t,\delta \right) & = & \lim_{N\to \infty }m_{h}^{f_{\infty }}\left(Z,t,N,\delta \right),\\ h_{Z}\left(f_{\infty },\delta \right) & = & \inf\{t:m_{h}^{f_{\infty }}\left(Z,t,\delta \right)=0\},\\ h_{top}\left(f_{\infty },Z\right) & = & \lim_{\delta \to 0}h_{Z}\left(f_{\infty },\delta \right). \end{array}$$

The quantity $h_{top}\left(f_{\infty },Z\right)$ is called the *topological entropy* of the dynamical solenoid $\left(X_{\infty },f_{\infty }\right)$ restricted to the set *Z*.

Let $0_{\infty }$ denote the multipotential which is the sequence of zero functions. By previous definitions, we immediately obtain

$$m_{h}^{f_{\infty }}\left(Z,t,N,\delta \right)=m_{P}\left(Z,t,0_{\infty },N,\delta \right).$$

Consequently,

(9) $$P_{Z}\left(0_{\infty }\right)=h_{top}\left(f_{\infty },Z\right).$$

**Proposition** **3.** *Let α,β∈R and α≤β. Assume that for any x∈Z,*

(10) $$\alpha \leq \underset{n\to \infty }{lim inf}\frac{1}{n}\left(S_{n}^{f_{\infty }}\varphi_{\infty }\right)\left(x\right)\leq \underset{n\to \infty }{lim sup}\frac{1}{n}\left(S_{n}^{f_{\infty }}\varphi_{\infty }\right)\left(x\right)\leq \beta .$$

*Then*

(a) *For h>0 and t∈R,*
$$P_{Z}\left(t\varphi_{\infty }\right)+\alpha h\leq P_{Z}\left(\left(t+h\right)\varphi_{\infty }\right)\leq P_{Z}\left(t\varphi_{\infty }\right)+\beta h.$$
(b) *For h<0 and t∈R,*
$$P_{Z}\left(t\varphi_{\infty }\right)+\beta h\leq P_{Z}\left(\left(t+h\right)\varphi_{\infty }\right)\leq P_{Z}\left(t\varphi_{\infty }\right)+\alpha h.$$

**Proof.**  Let ε>0. For any natural m≥1, let

$$Z_{m}=\underset{n\geq m}{⋂}\left\{z\in Z:\alpha -\varepsilon <\frac{1}{n}\left(S_{n}^{f_{\infty }}\varphi_{\infty }\right)\left(z\right)<\beta +\varepsilon \right\}.$$

Directly, for the definition, ${\left(Z_{m}\right)}_{m\geq 1}$ is an increasing sequence of sets, i.e., $Z_{m}\subset Z_{m+1}$. Moreover, $Z={⋃}_{m\geq 1}Z_{m}$. The inclusion “⊃" follows directly from the definition of sets $Z_{m}$. However, the second inclusion “⊂" follows from the properties of the limit inferior and limit superior of the sequence.

This implies that, by Lemma 10,

(11) $$P_{Z}\left(\varphi_{\infty }\right)=\sup\left\{P_{Z_{m}}\left(\varphi_{\infty }\right):m\geq 1\right\}.$$

(a) Fix t∈R and h>0. For N≥m and $I\in I(Z_{m},N,\delta )$ we have the following estimations:

$$\begin{array}{cc} \Phi (s,I,\left(t+h\right)\varphi_{\infty }) & =\Phi \left(s,I,\left(t+h\right),\varphi_{\infty }\right)\geq \Phi \left(s-h\left(\alpha -\varepsilon \right),t\varphi_{\infty }\right),\\ \Phi (s,I,\left(t+h\right)\varphi_{\infty }) & =\Phi \left(s,I,\left(t+h\right),\varphi_{\infty }\right)\leq \Phi \left(s-h\left(\beta +\varepsilon \right),t\varphi_{\infty }\right). \end{array}$$

This implies the estimations

$$\begin{array}{cc} m_{P}\left(Z_{m},s-h\left(\alpha -\varepsilon \right),t\varphi_{\infty },N,\delta \right) & \leq m_{P}\left(Z_{m},s,\left(t+h\right)\varphi_{\infty },N,\delta \right)\\ \leq & m_{P}\left(Z_{m},s-h\left(\beta +\varepsilon \right),t\varphi_{\infty },N,\delta \right),\\ m_{P}\left(Z_{m},s-h\left(\alpha -\varepsilon \right),t\varphi_{\infty },\delta \right)\leq & m_{P}\left(Z_{m},s,\left(t+h\right)\varphi_{\infty },\delta \right)\\ \leq & m_{P}\left(Z_{m},s-h\left(\beta +\varepsilon \right),t\varphi_{\infty },\delta \right). \end{array}$$

If the points $s^{\prime }$ and $s^{\prime \prime }$ coincide with critical points of functions

$$\begin{array}{cc} s & \mapsto m_{P}\left(Z_{m},s-h\left(\alpha -\varepsilon \right),t\varphi_{\infty },\delta \right),\\ s & \mapsto m_{P}\left(Z_{m},s-h\left(\beta +\varepsilon \right),t\varphi_{\infty },\delta \right), \end{array}$$

then

$$\begin{array}{cc} s^{\prime }\leq P_{Z_{m}}\left(\left(t+h\right)\varphi_{\infty },\delta \right), & \;s^{\prime }-h\left(\alpha -\varepsilon \right)=P_{Z_{m}}\left(t\varphi_{\infty },\delta \right),\\ s^{\prime \prime }\geq P_{Z_{m}}\left(\left(t+h\right)\varphi_{\infty },\delta \right), & \;s^{\prime \prime }-h\left(\beta +\varepsilon \right)=P_{Z_{m}}\left(t\varphi_{\infty },\delta \right), \end{array}$$

which leads to the following estimations:

$$\begin{array}{cc} P_{Z_{m}}\left(t\varphi_{\infty },\delta \right)+h\left(\alpha -\varepsilon \right) & \leq P_{Z_{m}}\left(\left(t+h\right)\varphi_{\infty },\delta \right)\\ \; & \leq P_{Z_{m}}\left(t\varphi_{\infty },\delta \right)+h\left(\beta +\varepsilon \right),\\ P_{Z_{m}}\left(t\varphi_{\infty }\right)+h\left(\alpha -\varepsilon \right) & \leq P_{Z_{m}}\left(\left(t+h\right)\varphi_{\infty }\right)\\ \; & \leq P_{Z_{m}}\left(t\varphi_{\infty }\right)+h\left(\beta +\varepsilon \right). \end{array}$$

Hence, by (11), passing to the limit with ε→0, we obtain (a).

The proof of (b) is analogous to the proof of item (a). □

**Remark** **1.** *The estimation in Proposition 3(b) can be obtained by (a) by taking t−h instead of t. We can do this because t is an arbitrary real number. Then, for h>0, we obtain*

$$P_{Z}\left(\left(t-h\right)\varphi_{\infty }\right)+\alpha h\leq P_{Z}\left(t\varphi_{\infty }\right)\leq P_{Z}\left(\left(t-h\right)\varphi_{\infty }\right)+\beta h,$$

*which is equivalent to the estimation*

(12) $$\begin{array}{cc} P_{Z}\left(t\varphi_{\infty }\right)+\beta \left(-h\right) & \leq P_{Z}\left(\left(t+\left(-h\right)\right)\varphi_{\infty }\right)\\ & \leq P_{Z}\left(t\varphi_{\infty }\right)+\alpha \left(-h\right),\;h>0. \end{array}$$

*Inputting (12) −h instead of h, we obtain Proposition 3(b).*

Notice that

$$\begin{array}{cc} \underset{n\to \infty }{lim inf}\frac{1}{n}S_{n}^{f_{\infty }}\left(-\log a_{\infty }\right) & =\underset{n\to \infty }{lim inf}\frac{1}{n}\left(-S_{n}^{f_{\infty }}\left(\log a_{\infty }\right)\right)\\ \; & =-\underset{n\to \infty }{lim sup}\frac{1}{n}S_{n}^{f_{\infty }}\left(\log a_{\infty }\right)=-\bar{\lambda^{f_{\infty }}},\\ \underset{n\to \infty }{lim sup}\frac{1}{n}S_{n}^{f_{\infty }}\left(-\log a_{\infty }\right) & =\underset{n\to \infty }{lim sup}\frac{1}{n}\left(-S_{n}^{f_{\infty }}\left(\log a_{\infty }\right)\right)\\ \; & =-\underset{n\to \infty }{lim inf}\frac{1}{n}S_{n}^{f_{\infty }}\left(\log a_{\infty }\right)=-\underline{\lambda^{f_{\infty }}}. \end{array}$$

Therefore, taking $\varphi_{\infty }=-\log a_{\infty }$ in Proposition 3, we obtain that the condition (10) is equivalent to the following:

(13) $$-\beta \leq \underline{\lambda^{f_{\infty }}}\left(x\right)\leq \bar{\lambda^{f_{\infty }}}\left(x\right)\leq -\alpha ,\;x\in Z.$$

**Corollary** **6.** *Let c,C∈R with c≤C. Assume that for any x∈Z,*

$$c\leq \underline{\lambda^{f_{\infty }}}\left(x\right)\leq \bar{\lambda^{f_{\infty }}}\left(x\right)\leq C.$$

*Then*

(a) *For h>0 and t∈R,*
$$P_{Z}\left(-t\log a_{\infty }\right)-Ch\leq P_{Z}\left(-\left(t+h\right)\log a_{\infty }\right)\leq P_{Z}\left(-t\log a_{\infty }\right)-ch.$$
(b) *For h<0 and t∈R,*
$$P_{Z}\left(-t\log a_{\infty }\right)-ch\leq P_{Z}\left(-\left(t+h\right)\log a_{\infty }\right)\leq P_{Z}\left(-t\log a_{\infty }\right)-Ch.$$
(c) *If c>0, the pressure function $t\mapsto P_{Z}\left(-t\log a_{\infty }\right)$ is a continuous, strictly decreasing function with Lipschitz constant C.*
(d) *If C<0, the pressure function $t\mapsto P_{Z}\left(-t\log a_{\infty }\right)$ is a continuous, strictly increasing function with Lipschitz constant |c|.*
(e) *If c=C, i.e., the Lyapunov exponent $\lambda^{f_{\infty }}$ is constant on Z, then the pressure function $t\mapsto P_{Z}\left(-t\log a_{\infty }\right)$ reduces to a linear function of form $t\mapsto h_{top}\left(f_{\infty },Z\right)-\lambda^{f_{\infty }}\cdot t$. In particular, if c=C=0, then the pressure function is constant and equal to $h_{top}\left(f_{\infty },Z\right)$.*

**Proof.**  Estimations (a) and (b) are a direct consequence of the condition (13) and Proposition 3; where we take take α=−C and β=−c. (c) Since c>0, for h>0 we obtain ch>0. Hence, by (a),

$$P_{Z}\left(-\left(t+h\right)\log a_{\infty }\right)\leq P_{Z}\left(-t\log a_{\infty }\right)-ch<P_{Z}\left(-t\log a_{\infty }\right),$$

which implies that the function $t\mapsto P_{Z}\left(-t\log a_{\infty }\right)$ is strictly decreasing. Thus, by (a), we obtain

$$0<P_{Z}\left(-t\log a_{\infty }\right)-P_{Z}\left(-\left(t+h\right)\log a_{\infty }\right)\leq Ch,\;t>0.$$

Consequently,

$$|P_{Z}\left(-t_{1}\log a_{\infty }\right)-P_{Z}\left(-t_{2}\log a_{\infty }\right)\left|\leq C\right|t_{1}-t_{2}|,\;t_{1},t_{2}\in R.$$

Therefore, the function $t\mapsto P_{Z}\left(-t\log a_{\infty }\right)$ is a continuous with Lipschitz constant *C*.

(d) Since C<0, for h>0, −Ch=|C|h>0. Hence, by (a),

$$P_{Z}\left(-\left(t+h\right)\log a_{\infty }\right)\geq P_{Z}\left(-t\log a_{\infty }\right)+\left|C\right|h>P_{Z}\left(-t\log a_{\infty }\right),$$

which implies that the function $t\mapsto P_{Z}\left(-t\log a_{\infty }\right)$ is strictly increasing. Thus, by (a), we obtain

$$0<P_{Z}\left(-\left(t+h\right)\log a_{\infty }\right)-P_{Z}\left(-t\log a_{\infty }\right)\leq -ch=\left|c\right|h,\;t>0.$$

Consequently,

$$|P_{Z}\left(-t_{1}\log a_{\infty }\right)-P_{Z}\left(-t_{2}\log a_{\infty }\right)\left|\leq |c|\right|t_{1}-t_{2}|,\;t_{1},t_{2}\in R,$$

which yields the Lipschitz continuity of the function.

(e) Observe first that $P_{Z}\left(0\log a_{\infty }\right)=P_{Z}\left(0_{\infty }\right)=h_{top}\left(f_{\infty },Z\right)$, by (9). The equality c=C implies that $c=C=\lambda^{f_{\infty }}$ and $\lambda^{f_{\infty }}$ is constant on *Z*. Combining (a) and (b), we conclude that for any t,h∈R,

$$P_{Z}\left(-t\log a_{\infty }\right)-\lambda^{f_{\infty }}h=P_{Z}\left(-\left(t+h\right)\log a_{\infty }\right).$$

Substituting h=−t we obtain

$$P_{Z}\left(-t\log a_{\infty }\right)+\lambda^{f_{\infty }}t=P_{Z}\left(-0\log a_{\infty }\right)=P_{Z}\left(0_{\infty }\right)=h_{top}\left(f_{\infty },Z\right).$$

Hence, $P_{Z}\left(-t\log a_{\infty }\right)=h_{top}\left(f_{\infty },Z\right)-\lambda^{f_{\infty }}t$. □

### 3.4. Proof of Theorem 3

**Proof.**  Let

$$h_{1}=\frac{h_{top}\left(f_{\infty },Z\right)}{C},\;h_{2}=\frac{h_{top}\left(f_{\infty },Z\right)}{c}.$$

Observe first that $h_{1}\leq h_{2}$ whenever 0<c≤C or c≤C<0. We know by Corollary 6(c) and (d) that the pressure function $t\mapsto P_{Z}\left(-t\log a_{\infty }\right)$ is continuous and strictly increasing for C<0, while it is strictly decreasing for c>0. In both cases, it follows that there exists at most one root $t^{\prime }\in \left[h_{1},h_{2}\right]$ such that $P_{Z}\left(-t^{\prime }\log a_{\infty }\right)=0$.

Consequently, to prove the assertion, it suffices to show that the pressure function has different signs at the ends of the interval $\left[h_{1},h_{2}\right]$, or, equivalently,

(14) $$P_{Z}\left(-h_{1}\log a_{\infty }\right)P_{Z}\left(-h_{2}\log a_{\infty }\right)\leq 0.$$

If 0<c≤C, then $h_{1}\leq h_{2}$. Moreover, by Corollary 6(a) with t=0, we obtain

$$P_{Z}\left(-h_{1}\log a_{\infty }\right)\geq 0,\;P_{Z}\left(-h_{2}\log a_{\infty }\right)\leq 0;$$

thus, (14) follows.

If c≤C<0, then $h_{1}\leq h_{2}$. Moreover, by Corollary 6(b) with t=0, we obtain

$$P_{Z}\left(-h_{1}\log a_{\infty }\right)\leq 0,\;P_{Z}\left(-h_{2}\log a_{\infty }\right)\geq 0;$$

thus, (14) follows. The proof is complete. □

## 4. Hausdorff Dimension

Let us recall the definition of the Hausdorff measure and dimension. Let (Y,d) be a metric space and Z⊂Y. Let δ>0. A family V of subsets of the space *Y* is called a *countable δ-cover of the set Z* if

(1) The family V consists of at most countably many sets.
(2) The family V covers the set *Z*, i.e., Z⊂⋃V.
(3) The diameter of each set in the family V is <δ, i.e., for any V∈V, diamV<δ.

The family of all countable δ-covers of the set *Z* is denoted by D(Z,δ). For real numbers s≥0 and δ>0, let us define

$$H^{s}\left(Z,\delta \right)=\inf\left\{\sum_{V\in V}{\left[\mathrm{diam}\;\left(V\right)\right]}^{s}:V\in D\left(Z,\delta \right)\right\}.$$

The mapping $\delta \mapsto H^{s}\left(Z,\delta \right)$ is monotonically increasing with respect to δ. Therefore, there exists a limit

$$H^{s}\left(Z\right)=\lim_{\delta \to 0}H^{s}\left(Z,\delta \right)\in \left[0,+\infty \right],$$

which is called the *s-dimensional Hausdorff measure* of the set *Z*. The graph of the function $s\mapsto H^{s}\left(Z\right)$ is symbolically illustrated below.

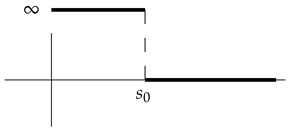



The function has exactly one critical point $s_{0}$ where it “drops” from infinity to zero. The critical point $s_{0}$ is called the *Hausdorff dimension* of the set *Z* and is denoted by $\dim_{H}\left(Z\right)$, i.e.,

$$\begin{array}{cc} \dim_{H}\left(Z\right) & =\inf\left\{s\geq 0:H^{s}\left(Z\right)=0\right\}\\ & =\sup\left\{s\geq 0:H^{s}\left(Z\right)=+\infty \right\}. \end{array}$$

One may equivalently define Hausdorff measure and Hausdorff dimension using covers by open balls; to this definition, we will refer in the proof of Theorem 4. For a subset Z⊂Y, let us define

$$\begin{array}{cc} M_{H}\left(Z,t,r\right)\; & =\inf\left\{\sum_{(x_{i},r_{i})\in I}{\left(2\cdot r_{i}\right)}^{t}:Z\subset \underset{(x_{i},r_{i})\in I}{⋃}B\left(x_{i},r_{i}\right),\;\;r_{i}<r,\;\;\;\;x_{i}\in Z\right\}, \end{array}$$

where *I* is a set of at most countably many elements. The mapping $r\mapsto M_{H}\left(Z,t,r\right)$ is monotonic with respect to *r*. Therefore, there exists a limit

$$M_{H}\left(Z,t\right)=\lim_{r\to 0}M_{H}\left(Z,t,r\right)\in \left[0,+\infty \right].$$

Varying t>0, we obtain a monotonic function $t\mapsto M_{H}\left(Z,t\right)$. This function has exactly one critical point, denoted by $\dim_{H}^{*}\left(Z\right)$, where it “drops" from infinity to zero, i.e.,

$$\begin{array}{ccc} \dim_{H}^{*}\left(Z\right) & = & \inf\left\{t>0:M_{H}\left(Z,t\right)=0\right\}\\ & = & \sup\left\{t>0:M_{H}\left(Z,t\right)=+\infty \right\}. \end{array}$$

**Proposition** **4.** *For any subset Z of a metric space (Y,d), the following equality holds:*

$$\dim_{H}\left(Z\right)=\dim_{H}^{*}\left(Z\right).$$

**Proof.**  See [12] the proof of Proposition 5.1. □

## 5. Properties of Dynamical Balls of DCSE-Solenoids

We say that a regular dynamical conformal solenoid $\left(X_{\infty },f_{\infty },a_{\infty }\right)$ is *simple and expanding* (in short: DCSE-solenoid) if

(1) Each map $f_{n}$ is a strong conformal map with strong dilatation $a_{n}$.
(2) There exists K>1 such that for any n≥1 and any x∈X, $1<a_{n}\left(x\right)<K$.
(3) The sequence $f_{\infty }$ consists of only finitely many distinct maps.

We will show that Bowen’s formula holds for DCSE-solenoids.

**Lemma** **11.** *Let f be a strong regular conformal map with strong dilatation a>0 defined on a compact metric space (X,d). We define*

$$A_{f}\left(x,y\right)=\left\{\begin{array}{cc} \log d(f(x),f(y))-\log d(x,y), & \mathrm{when}\;x\neq y\\ \log a(x), & \mathrm{when}\;x=y. \end{array}\right.$$

*Then, there exists a neighborhood $U_{f}$ of the diagonal Δ⊂X×X, such that $A_{f}$ is well defined and uniformly continuous on $U_{f}$.*

**Proof.**  A direct consequence of Proposition 2. □

**Corollary** **7.** *Assume that $\left(X_{\infty },f_{\infty },a_{\infty }\right)$ is a DCSE-solenoid. Then,*

(a) *There exists a neighborhood U of the diagonal Δ⊂X×X, such that the sequence $\left(A_{f_{n}}\right)$ is well defined and uniformly continuous in U.*
(b) *For any ε>0, there exists $\delta^{\prime }>0$ such that for any natural number n≥1,*
$$d\left(f_{n}\left(x\right),f_{n}\left(y\right)\right)e^{-(\log a_{n}\left(x\right)+\varepsilon )}<d\left(x,y\right)<d\left(f_{n}\left(x\right),f_{n}\left(y\right)\right)e^{-(\log a_{n}\left(x\right)-\varepsilon )},$$
  *whenever x,y∈X and $d\left(x,y\right)<\delta^{\prime }$.*

**Proof.**  (a) By the definition of a DCSE-solenoid, there exists a natural number k≥1 such that for any natural number *n*,

$$f_{n}\in \left\{f_{1},\ldots ,f_{k}\right\}.$$

Let $U_{i}=U_{f_{i}}$ be the neighborhood of the diagonal Δ, determined by Lemma 11, for each i=1,…,k. Set $U=U_{1}\cap U_{2}\cap \cdots \cap U_{k}$. In the neighborhood U, the sequence $\left(A_{f_{n}}\right)$ is well defined and uniformly continuous since it consists of a finite number of different maps. Thus, $\left(A_{f_{n}}\right)$ is uniformly continuous in U.

(b) Choose $\delta_{U}>0$, determined by Lemma 4, for the neighborhood U. Let ε>0. From the equality $\tilde{d}\left(\left(x,y\right),\left(x,x\right)\right)=d\left(x,y\right)$ (see the proof of Lemma 4) and the uniform continuity of the sequence $\left(A_{f_{n}}\right)$, there exists a positive $\delta^{\prime }<\delta_{U}$ such that for any n≥1

(15) $$|A_{f_{n}}\left(x,y\right)-A_{f_{n}}\left(x,x\right)|<\varepsilon ,$$

whenever d(x,y)<δ. Directly from the definition of the function $A_{f_{n}}$, inequality (15) is equivalent to the double inequality

$$\log d\left(f_{n}\left(x\right),f_{n}\left(y\right)\right)-\log a_{n}\left(x\right)-\varepsilon <\log d\left(x,y\right)<\log d\left(f_{n}\left(x\right),f_{n}\left(y\right)\right)-\log a_{n}\left(x\right)+\varepsilon .$$

Taking the exponential function exp to all sides of the double inequality, we complete the proof. □

**Lemma** **12.** *Assume that $\left(X_{\infty },f_{\infty },a_{\infty }\right)$ is a DCSE-solenoid. Then, for any ε>0, there exists $\delta^{\prime }>0$ such that for any x∈X and any natural number n≥1*

$$B\left(x,\delta \cdot \exp\left(-n\left(\lambda_{n}^{f_{\infty }}\left(x\right)+\varepsilon \right)\right)\right)\subset B\left(x,n,\delta \right)\subset B\left(x,\delta \cdot \exp\left(-n\left(\lambda_{n}^{f_{\infty }}\left(x\right)-\varepsilon \right)\right)\right),$$

*whenever $0<\delta <\delta^{\prime }$.*

**Proof.**  Take ε>0. Choose $\delta^{\prime }>0$ determined by Corollary 7 (b). Let $0<\delta <\delta^{\prime }$ and n≥1. For any point z∈X, let $z_{i}=f_{i,n}\left(z\right)$, for i=0,…, n.

In the first step, we intend to prove the inclusion $B\left(x,n,\delta \right)\subset B\left(x,\delta \cdot e^{-n(\lambda_{n}^{f_{\infty }}\left(x\right)-\varepsilon )}\right)$. Consider the dynamical ball

$$B\left(x,n,\delta \right)=\{y\in X:\left(d\left(x_{i},y_{i}\right)<\delta ,\;i=0,\ldots ,n\right\}.$$

Let x∈X and y∈B(x,n,δ). Then $d(x_{i},y_{i})<\delta$, which means that the second inequality of Corollary 7(b) can be rewritten as follows:

$$d\left(x_{i},y_{i}\right)<d\left(f_{i}\left(x_{i}\right),f_{i}\left(y_{i}\right)\right)e^{-(\log a_{i}\left(x_{i}\right)-\varepsilon )},$$

i.e., due to the equalities $x_{i-1}=f_{i}\left(x_{i}\right)$ and $y_{i-1}=f_{i}\left(y_{i}\right)$, we obtain

$$d\left(x_{i},y_{i}\right)<d\left(x_{i-1},y_{i-1}\right)e^{-(\log a_{i}\left(x_{i}\right)-\varepsilon )}.$$

Consequently, using the definition of $\lambda_{i,j}^{f_{\infty }}$ (see Section 3) for y∈B(x,n,δ), we successively obtain the following.

For n≥1,

$$\begin{array}{cc} d(x,y) & =d\left(x_{n},y_{n}\right)<d\left(x_{n-1},y_{n-1}\right)\cdot e^{-(\log a_{n}\left(x_{n}\right)-\varepsilon )}\\ \; & =d\left(x_{n-1},y_{n-1}\right)\cdot \exp\left(-(\lambda_{n,n}^{f_{\infty }}\left(x\right)-\varepsilon )\right). \end{array}$$

For n≥2,

$$\begin{array}{cc} d(x,y) & =d\left(x_{n},y_{n}\right)<d\left(x_{n-1},y_{n-1}\right)\cdot e^{-(\log a_{n}\left(x_{n}\right)-\varepsilon )}\\ \; & <d\left(x_{n-2},y_{n-2}\right)\cdot e^{-(\log a_{n-1}\left(x_{n-1}\right)-\varepsilon )}\cdot e^{-(\log a_{n}\left(x_{n}\right)-\varepsilon )}\\ \; & =d\left(x_{n-2},y_{n-2}\right)\cdot e^{-(\log a_{n-1}\left(x_{n-1}\right)+\log a_{n}\left(x_{n}\right)-2\varepsilon )}\\ \; & =d\left(x_{n-2},y_{n-2}\right)\cdot \exp\left(-2(\lambda_{n-1,n}^{f_{\infty }}\left(x\right)-\varepsilon )\right). \end{array}$$

For n≥j,

$$d\left(x,y\right)<d\left(x_{n-j},y_{n-j}\right)\cdot \exp\left(-j\cdot (\lambda_{n-j+1,n}^{f_{\infty }}\left(x\right)-\varepsilon )\right).$$

Taking j=n and applying the equality $\lambda_{1,n}^{f_{\infty }}=\lambda_{n}^{f_{\infty }}$ we obtain

$$d\left(x,y\right)<d\left(x_{0},y_{0}\right)\cdot \exp\left(-n\cdot (\lambda_{n}^{f_{\infty }}\left(x\right)-\varepsilon )\right)<\delta \cdot \exp\left(-n\cdot (\lambda_{n}^{f_{\infty }}\left(x\right)-\varepsilon )\right),$$

which means that $y\in B\left(x,\delta \cdot \exp\left(-n\cdot (\lambda_{n}^{f_{\infty }}\left(x\right)-\varepsilon )\right)\right)$.

In the second step, we are going to prove the inclusion $B\left(x,\delta \cdot e^{-n(\lambda_{n}^{f_{\infty }}\left(x\right)+\varepsilon )}\right)\subset B\left(x,n,\delta \right)$. Since the dynamical solenoid is expanding, $\log a_{i}>0$, for any i≥1. In particular, it follows that for any 1≤i≤j≤n we have $\left(n-i+1\right)\lambda_{i,n}^{f_{\infty }}\geq \left(n-j+1\right)\lambda_{j,n}^{f_{\infty }}$. Setting i=1, we obtain

(16) $$\delta e^{-n(\lambda_{n}^{f_{\infty }}+\varepsilon )}\leq \delta e^{-\left(n-j+1\right)\left(\lambda_{j,n}^{f_{\infty }}+\varepsilon \right)}<\delta ,\;\mathrm{for}\;j=1,\ldots ,n.$$

We fix x∈X and consider a point $y\in B(x,\delta e^{-n(\lambda_{n}^{f_{\infty }}\left(x\right)+\varepsilon )})$. Applying the identities $x_{n}=x$, $y_{n}=y$ and definition of B(x,n,δ), we have

$$d\left(x_{n},y_{n}\right)=d\left(x,y\right)<\delta e^{-n(\lambda_{n}^{f_{\infty }}\left(x\right)+\varepsilon )}.$$

Due to (16), for any j=1,…,n, we obtain the inequality

$$d\left(x_{n},y_{n}\right)<\delta e^{-\left(n-j+1\right)\left(\lambda_{j,n}^{f_{\infty }}+\varepsilon \right)}.$$

From the first inequality of Corollary 7 (b), we successively obtain the following.

For n≥1,

$$\begin{array}{ccc} d(x_{n-1},y_{n-1}) & = & d(f_{n}\left(x_{n}\right),f_{n}\left(y_{n}\right))\\ & < & d\left(x_{n},y_{n}\right)\cdot e^{\lambda_{n,n}^{f_{\infty }}\left(x_{n}\right)+\varepsilon }\\ & = & d\left(x_{n},y_{n}\right)e^{\lambda_{n,n}^{f_{\infty }}\left(x_{n}\right)+\varepsilon }<\delta . \end{array}$$

For n≥2,

$$\begin{array}{ccc} d(x_{n-2},y_{n-2}) & = & d(f_{n-1}\left(x_{n-1}\right),f_{n-1}\left(y_{n-1}\right))\\ & < & d\left(x_{n-1},y_{n-1}\right)\cdot e^{a_{n-1}\left(x_{n-1}\right)+\varepsilon }\\ & = & d\left(x_{n},y_{n}\right)\cdot e^{a_{n}\left(x_{n}\right)+\varepsilon }\cdot e^{a_{n-1}\left(x_{n-1}\right)+\varepsilon }\\ & = & d\left(x_{n},y_{n}\right)\cdot e^{2(\lambda_{n-1,n}^{f_{\infty }}\left(x_{n}\right)+\varepsilon )}<\delta . \end{array}$$

For n≥j,

$$\begin{array}{cc} d(x_{n-j},y_{n-j}) & =d(f_{n-j+1}\left(x_{n-j+1}\right),f_{n-j+1}\left(y_{n-j+1}\right))\\ \; & <d\left(x_{n-j+1},y_{n-j+1}\right)\cdot e^{a_{n-j+1}\left(x_{n-j+1}\right)+\varepsilon }\\ \; & =d\left(x_{n},y_{n}\right)\cdot e^{a_{n}\left(x_{n}\right)+\varepsilon }\cdots e^{a_{n-j+1}\left(x_{n-j+1}\right)+\varepsilon }\\ \; & =d\left(x_{n},y_{n}\right)\cdot e^{j(\lambda_{n-j+1,n}^{f_{\infty }}\left(x_{n}\right)+\varepsilon )}<\delta . \end{array}$$

We have shown that for any k=0,…,n the inequality $d(x_{k},y_{k})<\delta$ holds, which is equivalent to y∈B(x,n,δ). □

## 6. Proof of Theorem  4

Let $t^{*}>0$ denote the unique root of the pressure function $t\mapsto P_{Z}\left(-t\cdot \log a_{\infty }\right)$, which exists and is positive by Theorem 3. Hence, by Corollary 6 (c), we have

(17) $$\begin{array}{ccc} P_{Z}\left(-t\cdot \log a_{\infty }\right) & < & 0,\;\mathrm{for}\;t>t^{*} \end{array}$$

(18) $$\begin{array}{ccc} P_{Z}\left(-t\cdot \log a_{\infty }\right) & > & 0,\;\mathrm{for}\;t<t^{*} \end{array}$$

First, we will show that $\dim_{H}\left(Z\right)\leq t^{*}$. For a natural number m∈N and α>0, let us define a set

$$Z_{m}=\left\{x\in Z:\lambda_{n}^{f_{\infty }}\left(x\right)>\alpha ,\mathrm{for}\;\mathrm{any}\;n\geq m\right\}.$$

Since $\left(X_{\infty },f_{\infty },a_{\infty }\right)$ is a DCSE-solenoid, there exists β>0 such that $\bar{\lambda^{f_{\infty }}}<\beta$. Note (cf. Section 3) that

$$A\left(\left(\alpha ,\beta \right)\right)=\left\{x\in X:\alpha <\underline{\lambda^{f_{\infty }}}\left(x\right)\leq \bar{\lambda^{f_{\infty }}}\left(x\right)<\beta \right\}.$$

Directly from the definition of $Z_{m}$ and the inclusion Z⊂A((α,β)), we obtain

(19) $$Z_{m}\subset Z_{m+1},\;\underset{m\in N}{⋃}Z_{m}=Z.$$

We fix $t>t^{*}\geq 0$. Due to (17), we can choose 0<ε<α, such that

(20) $$\begin{array}{c} -t\cdot \varepsilon >P_{Z}\left(-t\cdot \log a_{\infty }\right)\geq P_{Z_{m}}\left(-t\cdot \log a_{\infty }\right), \end{array}$$

where the second inequality is a consequence of Lemma 9. By Lemma 12, there exists $\delta^{\prime }>0$ such that for any $x\in Z_{m}$, n≥m, and any $0<\delta <\delta^{\prime }$,

(21) $$\begin{array}{cc} \mathrm{diam}\;B(x,n,\delta ) & \leq 2\delta \exp(-n\left(\lambda_{n}^{f_{\infty }}\left(x\right)-\varepsilon \right))\\ & <2\delta \exp(-n(\alpha -\varepsilon )). \end{array}$$

This implies that for any N>m and any $0<\delta <\delta^{\prime }$,

(22) $$P\left(Z_{m},N,\delta \right)\subset D\left(Z_{m},2\delta \exp\left(-N\left(\alpha -\varepsilon \right)\right)\right).$$

Note that the first inequality in (21) leads to the estimation

$${\left[\frac{1}{2\delta }\mathrm{diam}\;B\left(x,n,\delta \right)\right]}^{t}\leq \exp\left(-nt\left(\log\lambda_{n}^{f_{\infty }}\left(x\right)-\varepsilon \right)\right),\;n\geq m,x\in Z_{m}.$$

Therefore, for N>m and any $I\in I(Z_{m},N,\delta )$ applying definitions presented in Section 3, we have

$$\begin{array}{cc} \; & \Phi (-t\varepsilon ,-t\log a_{\infty })\\ & =\sum_{(x_{i},n_{i})\in I}\exp\left(-n_{i}t\left(\log\lambda_{n_{i}}^{f_{\infty }}\left(x_{i}\right)-\varepsilon \right)\right)\\ \; & \geq \sum_{(x_{i},n_{i})\in I}{\left[\frac{1}{2\delta }\mathrm{diam}\;B\left(x_{i},n_{i},\delta \right)\right]}^{t}\\ \; & \geq \inf\left\{\frac{1}{{\left(2\delta \right)}^{t}}\sum_{V\in V}{\left(\mathrm{diam}\;V\right)}^{t}:V\in D(Z_{m},2\delta \exp\left(-N\left(\alpha -\varepsilon \right)\right)\right\}\\ \; & =\frac{1}{{\left(2\delta \right)}^{t}}\cdot H^{t}\left(Z_{m},2\delta \exp\left(-N\left(\alpha -\varepsilon \right)\right)\right) \end{array}$$

This implies that

$$\begin{array}{cc} \; & m_{P}\left(Z_{m},-t\varepsilon ,-t\log a_{\infty },N,\delta \right)\\ & =\inf\{\Phi \left(-t\varepsilon ,-t\log a_{\infty },N,\delta \right):I\in I\left(Z_{m},N,\delta \right)\}\\ \; & \geq \frac{1}{{\left(2\delta \right)}^{t}}\cdot H^{t}\left(Z_{m},2\delta \exp\left(-N\left(\alpha -\varepsilon \right)\right)\right). \end{array}$$

Due to the inequality α−ε>0, in the limit with N→∞ yields

$$\begin{array}{cc} \; & m_{P}\left(Z_{m},-t\varepsilon ,-t\log a_{\infty },\delta \right)\\ & =\lim_{N\to \infty }m_{P}\left(Z_{m},-t\varepsilon ,-t\log a_{\infty },N,\delta \right)\\ \; & \geq \frac{1}{{\left(2\delta \right)}^{t}}\lim_{N\to \infty }H^{t}\left(Z_{m},2\delta \exp\left(-N\left(\alpha -\varepsilon \right)\right)\right)\\ \; & =\frac{1}{{\left(2\delta \right)}^{t}}\cdot H^{t}\left(Z_{m}\right). \end{array}$$

We claim that

(23) $$\begin{array}{c} H^{t}\left(Z_{m}\right)=0. \end{array}$$

Otherwise, if $H^{t}\left(Z_{m}\right)>0$ then by Lemma 8,

$$m_{P}\left(Z_{m},s,-t\log a_{\infty },\delta \right)=+\infty$$

for all real s<−tε. This means that

$$P_{Z_{m}}\left(-t\log a_{\infty },\delta \right)=\sup\left\{s\in R:m_{P}\left(Z_{m},s,-t\log a_{\infty },\delta \right)=+\infty \right\}\geq -t\varepsilon .$$

Then, by (8), we have

$$P_{Z_{m}}\left(-t\log a_{\infty }\right)=\sup\left\{P_{Z_{m}}\left(-t\log a_{\infty },\delta \right):\delta >0\right\}\geq -t\varepsilon ,$$

which contradicts the inequality (20). Thus, we have proven (23).

By properties of t−Hausdorff measure, we have

$$H^{t}\left(Z\right)=H^{t}\left(⋃Z_{m}\right)=\sup\left\{H^{t}\left(Z_{m}\right):m\geq 1\right\}=0,$$

which means that $\dim_{H}\left(Z\right)\leq t$ for any $t>t^{*}$. Thus, we obtain the inequality $\dim_{H}\left(Z\right)\leq t^{*}$.

Let us proceed to prove the inequality $\dim_{H}\left(Z\right)\geq t^{*}$. This inequality holds trivially when $t^{*}=0$. Therefore, assume that $t^{*}>0$. Take $0<t<t^{*}$. Then, due to (18), there exists ε>0 such that

$$0<t\varepsilon <P_{Z}\left(-t\log a_{\infty }\right).$$

Hence, by Lemma 5 and the definition of topological pressure, there is $\bar{\delta }>0$ such that for any $0<\delta <\bar{\delta }$,

(24) $$0<t\varepsilon <P_{Z}\left(-t\log a_{\infty },\delta \right).$$

Let $b=\sup\{a_{n}\left(x\right):x\in X,\;n\geq 1\}$. Since $\left(X_{\infty },f_{\infty },a_{\infty }\right)$ is a DCSE-solenoid, 1<b<∞. Notice that for any 1≤i≤j and any x∈X,

$$\lambda_{i,j}^{f_{\infty }}\left(x\right)=\frac{1}{j-i+1}\sum_{k=i}^{j}\log a_{k}\left(f_{k,j}\left(x\right)\right)\leq \frac{1}{j-i+1}\sum_{k=i}^{j}\log b=\log b.$$

For ε>0 chosen above, we fix $\delta^{\prime }>0$ given by Lemma 12. Let $0<\delta <\delta^{\prime }$. For 1≤i≤j, we set

$$s_{i,j}\left(x\right)=\delta \exp\left(-\left(j-i+1\right)\left(\lambda_{i,j}^{f_{\infty }}\left(x\right)+\varepsilon \right)\right).$$

In particular,

$$s_{1,n}\left(x\right)=\delta \exp\left(-n\left(\lambda_{1,n}^{f_{\infty }}+\varepsilon \right)\right)=\delta \exp\left(-n\left(\lambda_{n}^{f_{\infty }}\left(x\right)+\varepsilon \right)\right).$$

Since, for any natural n≥1, $\log a_{n}>0$, we obtain the estimation

$$s_{i,n}\left(x\right)<\delta \exp\left(-\left(n-i+1\right)\varepsilon \right)),\;\mathrm{for}\;n\geq i.$$

Hence, it follows that for any x∈X

(25) $$\lim_{n\to \infty }s_{i,n}\left(x\right)=0.$$

Moreover, this convergence is uniform. We have

$$\begin{array}{cc} \frac{s_{2,n}\left(x\right)}{s_{1,n}\left(x\right)} & =\frac{\delta \exp(-\left(n-1\right)\left(\lambda_{2,n}^{f_{\infty }}\left(x\right)+\varepsilon \right))}{\delta \exp(-n\left(\lambda_{1,n}^{f_{\infty }}\left(x\right)+\varepsilon \right))}\\ \; & =\frac{\exp(-S_{2,n}^{f_{\infty }}\left(\log a_{\infty }\right)\left(x\right)-\left(n-1\right)\varepsilon )}{\exp(-S_{1,n}^{f_{\infty }}\left(\log a_{\infty }\right)\left(x\right)-n\varepsilon )}\\ \; & =\exp\left[\left(-\sum_{k=2}^{n}\log a_{k}\left(f_{k,n}\left(x\right)\right)-\left(n-1\right)\varepsilon \right)\right.\\ \; & \;\;\;\left.-\left(-\sum_{k=1}^{n}\log a_{k}\left(f_{k,n}\left(x\right)\right)-n\varepsilon \right)\right]\\ \; & =\exp\left(\log a_{1}\left(f_{1,n}\left(x\right)\right)+\varepsilon \right). \end{array}$$

By the definition of *b*, we conclude that $\log b\geq \log(a_{1}\left(f_{1,n}\left(x\right)\right)$. Therefore,

$$1<\exp\left(\log a_{1}\left(f_{1,n}\left(x\right)\right)+\varepsilon \right)\leq \exp\left(\log b+\varepsilon \right).$$

Consequently we obtain

$$e^{-(\log b+\varepsilon )}s_{1,n}\left(x\right)<e^{-(\log b+\varepsilon )}s_{2,n}\left(x\right)\leq s_{1,n}\left(x\right)<s_{2,n}\left(x\right).$$

Hence, by (25), there exists $\bar{r}>0$ such that for any x∈Z and any $0<r<\bar{r}$, there exists n≥2, such that

$$\exp\left(-(\log b+\varepsilon )\right)s_{1,n}\left(x\right)\leq r<s_{1,n}\left(x\right)=\delta \exp\left(-n\left(\lambda_{n}^{f_{\infty }}\left(x\right)+\varepsilon \right)\right).$$

Thus,

$$\begin{array}{cc} r & \geq e^{-(\log b+\varepsilon )}s_{1,n}\left(x\right)\\ \; & =e^{-(\log b+\varepsilon )}\delta \exp\left(-n\left(\lambda_{n}^{f_{\infty }}\left(x\right)+\varepsilon \right)\right)\\ \; & \geq e^{-(\log b+\varepsilon )}\delta \exp\left(-n\left(\log b+\varepsilon \right)\right)\\ \; & =\delta \exp(-(n+1)(\log b+\varepsilon )), \end{array}$$

or, equivalently (after taking the logarithms of both sides),

n≥N(r,δ)andn∈N,

where

$$N\left(r,\delta \right)=\frac{-\log r+\log\delta }{\log b+\varepsilon }-1.$$

Note that the function r↦N(r,δ) is strictly decreasing with respect to r>0 and $\lim_{r\to 0}N\left(r,\delta \right)=+\infty$.

Let $0<r<\bar{r}$. Suppose that $\left\{B\left(x_{i},r_{i}\right):\left(x_{i},r_{i}\right)\in I\right\}$ is at most countable cover of the set *Z* with balls of positive radii $r_{i}<r$ and centers $x_{i}\in Z$. Then, due to previous considerations, there exists a sequence of natural numbers $\left(n_{i}\right)$ such that

$$\begin{array}{ccc} \delta \exp\left(-(\log b+\varepsilon )\right)s_{1,n_{i}}\left(x_{i}\right)\leq & r_{i} & <s_{1,n_{i}}\left(x_{i}\right),\\ N\left(r,\delta \right)<N\left(r_{i},\delta \right)\leq & n_{i}, \end{array}$$

where

$$s_{1,n_{i}}\left(x_{i}\right)=\delta \exp\left(-n_{i}\left(\lambda_{n_{i}}^{f_{\infty }}\left(x_{i}\right)+\varepsilon \right)\right).$$

Hence, by Lemma 12, we obtain the inclusion $B\left(x_{i},r_{i}\right)\subset B\left(x_{i},n_{i},\delta \right)$. This means that the cover $\left\{B\left(x_{i},r_{i}\right)\right\}$ is a refinement of the cover $\left\{B\left(x_{i},n_{i},\delta \right)\right\}$ of the set *Z* by dynamical balls. Since $\left\{B\left(x_{i},n_{i},\delta \right)\right\}\in P\left(Z,N\left(r,\delta \right),\delta \right)$, we proceed to the following estimations:

$$\begin{array}{cc} \; & M_{H}\left(Z,t,r\right)=\inf\left\{\sum_{(x_{i},r_{i})\in I}{\left(2r_{i}\right)}^{t}:Z\subset \underset{(x_{i},r_{i})\in I}{⋃}B\left(x_{i},r_{i}\right),\;\;r_{i}<r,\;\;\;\;x_{i}\in Z\right\}\\ \; & \geq \inf\left\{\sum_{(x_{i},r_{i})\in I}2^{t}e^{-(\log b+\varepsilon )t}s_{1,n_{i}}^{t}\left(x_{i}\right):Z\subset \underset{(x_{i},r_{i})\in I}{⋃}B\left(x_{i},r_{i}\right),\;\;r_{i}<r,\;\;\;\;x_{i}\in Z\right\}\\ \; & =\inf\left\{\sum_{(x_{i},r_{i})\in I}{\left(2\delta \right)}^{t}e^{-(\log b+\varepsilon )t}e^{-tn_{i}\left(\lambda_{n_{i}}^{f_{\infty }}\left(x_{i}\right)+\varepsilon \right)}:Z\subset \underset{(x_{i},r_{i})\in I}{⋃}B\left(x_{i},r_{i}\right),\;\;r_{i}<r,\;\;\;\;x_{i}\in Z\right\}\\ \; & ={\left(2\delta \right)}^{t}e^{-(\log b+\varepsilon )t}\inf\left\{\sum_{(x_{i},r_{i})\in I}e^{-tn_{i}\left(\lambda_{n_{i}}^{f_{\infty }}\left(x_{i}\right)+\varepsilon \right)}:Z\subset \underset{(x_{i},r_{i})\in I}{⋃}B\left(x_{i},r_{i}\right),\;\;r_{i}<r,\;\;\;\;x_{i}\in Z\right\}\\ \; & \geq {\left(2\delta \right)}^{t}e^{-(\log b+\varepsilon )t}\inf\left\{\Phi \left(t\varepsilon ,I,-t\log a_{\infty }\right):I\in I\left(Z,N\left(r,\delta \right),\delta \right)\right\}\\ \; & ={\left(2\delta \right)}^{t}e^{-(\log b+\varepsilon )t}m_{P}\left(Z,t\varepsilon ,-t\log a_{\infty },N\left(r,\delta \right),\delta \right). \end{array}$$

Therefore, we obtain

$$\begin{array}{cc} M_{H}\left(Z,t\right) & =\lim_{r\to 0}M_{H}\left(Z,t,r\right)\\ \; & \geq {\left(2\delta \right)}^{t}e^{-(\log b+\varepsilon )t}\lim_{r\to 0}m_{P}\left(Z,t\varepsilon ,-t\log a_{\infty },N\left(r,\delta \right),\delta \right)\\ \; & ={\left(2\delta \right)}^{t}e^{-(\log b+\varepsilon )t}m_{P}\left(Z,t\varepsilon ,-t\log a_{\infty },\delta \right)=+\infty , \end{array}$$

where the last equality follows from (5) and (7). Therefore, $\dim_{H}\left(Z\right)>t$. Since *t* was an arbitrary number from the interval $\left(0,t^{*}\right)$, we conclude that $\dim_{H}\left(Z\right)\geq t^{*}$. This completes the proof of the theorem.

Corollary 1 in Section 1 is a direct consequence of Theorem 4.

## 7. Apendix: Conformality vs. Strong Conformality

The following example compares dilatation with strong dilatation. On a plane, we consider four points: A(−2,0), B(2,0), O(0,0), Q(0,−2). Let $I_{1}$ be a segment AB, while $I_{2}$ a segment OQ. Consider a metric space $T=I_{1}\cup I_{2}$ equipped with the city metric d. By $|\bar{PR}|$, we mean the length of the segment PR. In particular, we have

(1) if $x\in \bar{AB}$, then $d\left(x,Q\right)=|\bar{xO}\left|+\right|\bar{OQ}\left|=\right|\bar{xO}|+2$;
(2) if $y\in \bar{OQ}$, then $d\left(y,Q\right)=|\bar{yQ}|$.

Let f:T→T be a homothety with ratio 1/2 and fixed point Q; it means that for any x∈T, there exists a unique point $f\left(x\right)\in \bar{OQ}$ with $d\left(f\left(x\right),Q\right)=\frac{1}{2}d\left(x,Q\right)$. Now we calculate a dilatation $a_{w}\left(x\right)$ for any x∈T. Notice that for x,y∈T and *f*, we have

$$d\left(f\left(x\right),Q\right)=\frac{1}{2}d\left(x,Q\right),\;d\left(f\left(y\right),Q\right)=\frac{1}{2}d\left(y,Q\right).$$

We fix x∈T. By the definition of (T,d), it easily follows that there exists a neighborhood Γ of *x* such that for any y∈Γ,

$$\begin{array}{cc} d(f(x),f(y)) & =|d(f(x),Q)-d(f(y),Q)|\\ & =\frac{1}{2}\left|d\left(x,Q\right)-d\left(y,Q\right)\right|=\frac{1}{2}d\left(x,y\right). \end{array}$$

Therefore, for any x∈T, we obtain

$$a_{w}\left(x\right)=\lim_{y\to x}\frac{d(f(x),f(y))}{d(x,y)}=\frac{1}{2},$$

so f:T→T is a conformal map. Consider a function of two variables ζ:T×T→R defined by the formula

$$\zeta \left(x,y\right)=\left\{\begin{array}{cc} \log d(f(x),f(y))-\log d(x,y), & \mathrm{if}\;x\neq y\\ \log a_{w}\left(x\right), & \mathrm{if}\;x=y. \end{array}\right.$$

We fix $x_{0}=\left(0,0\right)\in T$. By definition, the function ζ(x,y) is continuous at $\left(x_{0},x_{0}\right)$ if and only if for any sequences $x_{n}\to x_{0}$ and $y_{n}\to x_{0}$ the limit

$$\lim_{\begin{array}{c} x_{n}\to x_{0}\\ y_{n}\to x_{0} \end{array}}\zeta \left(x_{n},y_{n}\right)=\zeta \left(x_{0},x_{0}\right).$$

In particular, for the sequences $x_{n}=\left(-\frac{2}{n},0\right)$ and $y_{n}=\left(\frac{4}{n},0\right)$ we obtain $d\left(x_{n},y_{n}\right)=\frac{6}{n}$. Moreover, $f\left(x_{n}\right)=\left(0,\frac{1}{n}-1\right)$ and $f\left(y_{n}\right)=\left(0,\frac{2}{n}-1\right)$. Thus, $d\left(f\left(x_{n}\right),f\left(y_{n}\right)\right)=\frac{1}{n}$. Consequently,

$$\lim_{\begin{array}{c} x_{n}\to x_{0}\\ y_{n}\to x_{0} \end{array}}\zeta \left(x_{n},y_{n}\right)=-\log6.$$

However,

$$\zeta \left(x_{0},x_{0}\right)=\log a_{w}\left(x_{0}\right)=-\log2,$$

which yields discontinuity of ζ at the point $\left(x_{0},x_{0}\right)$. Moreover, the strong dilatation of the function f:T→T is not even defined at $x_{0}.$ It follows that f:T→T is an example of a conformal map which is not a strong conformal one.

Finally, we note that the strong conformality of *f* implies that $A_{f}:X\times X\to R$ is well defined and uniformly continuous on some neighborhood of the diagonal (see Lemma 11). This fact is a key step in the proof of crucial Lemma 12.

## Data Availability

No new data were created or analyzed in this study. Data sharing is not applicable to this article.
